# Poly[aqua­bis(μ-benzene-1,2-dicarboxyl­ato)ethano­ltetra­lithium]

**DOI:** 10.1107/S1600536809003390

**Published:** 2009-02-04

**Authors:** Patricia Rodríguez-Cuamatzi, Hugo Tlahuext, Herbert Höpfl

**Affiliations:** a Universidad Politécnica de Tlaxcala, Carretera Federal Tlaxcala-Puebla Km 9.5, Tepeyanco, Tlaxcala, México; b Centro de Investigaciones Químicas, Universidad Autónoma del Estado de Morelos, Av. Universidad 1001, Col. Chamilpa CP 62209, Cuernavaca Mor., México

## Abstract

In the crystal structure of the title compound [Li_4_(C_8_H_4_O_4_)_2_(C_2_H_5_OH)(H_2_O)]_*n*_, there are four crystallographically independent metal centers each of which is coordinated by four O atoms. The benzene-1,2-dicarboxyl­ate groups act as bidentate–bridging ligands producing a two-dimensional coordination network parallel to the *ab* plane. The coordination polymer is further stabilized by coordination of water and ethanol mol­ecules by the Li^+^ ions. Simultaneously, the water and ethanol mol­ecules are involved in O—H⋯O and C—H⋯π inter­actions.

## Related literature

For related literature, see: Łyszczek *et al.* (2008 ▶); Chae *et al.* (2004 ▶); García-Zarracino *et al.* (2003 ▶); García-Zarracino & Höpfl (2004 ▶); García-Zarracino *et al.* (2008 ▶). For analysis of hydrogen-bonding patterns, see: Hunter (1994 ▶); Desiraju (1991 ▶).
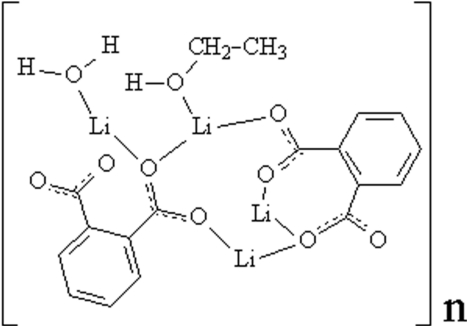


## Experimental

### 

#### Crystal data

[Li_4_(C_8_H_4_O_4_)_2_(C_2_H_6_O)(H_2_O)]
*M* *_r_* = 420.07
Triclinic, 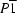

*a* = 7.5254 (7) Å
*b* = 10.0538 (10) Å
*c* = 13.5073 (13) Å
α = 106.460 (2)°
β = 91.185 (2)°
γ = 103.046 (2)°
*V* = 950.84 (16) Å^3^
*Z* = 2
Mo *K*α radiation
μ = 0.12 mm^−1^
*T* = 293 (2) K
0.49 × 0.36 × 0.06 mm

#### Data collection

Bruker SMART APEX CCD area-detector diffractometer
Absorption correction: multi-scan (*SADABS*; Sheldrick, 1996 ▶) *T*_min_ = 0.945, *T*_max_ = 1.000 (expected range = 0.938–0.993)
10069 measured reflections
3735 independent reflections
3110 reflections with *I* > 2σ(*I*)
*R*_int_ = 0.039

#### Refinement

*R*[*F* ^2^ > 2σ(*F* ^2^)] = 0.054
*wR*(*F* ^2^) = 0.129
*S* = 1.09
3735 reflections
299 parameters
3 restraints
H atoms treated by a mixture of independent and constrained refinement
Δρ_max_ = 0.29 e Å^−3^
Δρ_min_ = −0.20 e Å^−3^

### 

Data collection: *SMART* (Bruker, 2000 ▶); cell refinement: *SAINT-Plus-NT* (Bruker, 2001 ▶); data reduction: *SAINT-Plus-NT*; program(s) used to solve structure: *SHELXTL-NT* (Sheldrick, 2008 ▶); program(s) used to refine structure: *SHELXTL-NT*; molecular graphics: *SHELXTL-NT*; software used to prepare material for publication: *PLATON* (Spek, 2003 ▶) and *publCIF* (Westrip, 2009 ▶).

## Supplementary Material

Crystal structure: contains datablocks I, global. DOI: 10.1107/S1600536809003390/bt2860sup1.cif

Structure factors: contains datablocks I. DOI: 10.1107/S1600536809003390/bt2860Isup2.hkl

Additional supplementary materials:  crystallographic information; 3D view; checkCIF report

## Figures and Tables

**Table 1:** Hydrogen-bond geometry (Å, °)

*D*—H⋯*A*	*D*—H	H⋯*A*	*D*⋯*A*	*D*—H⋯*A*
O9—H9⋯O10^i^	0.841 (13)	1.947 (17)	2.767 (2)	165 (2)
O10—H10*A*⋯O1	0.84 (2)	1.97 (2)	2.764 (2)	156 (2)
O10—H10*B*⋯O6^i^	0.84 (1)	1.984 (12)	2.797 (2)	163 (3)
C17—H17*A*⋯*Cg*2	0.97	2.86	3.584 (3)	132
C18—H18*C*⋯*Cg*1^i^	0.96	2.70	3.572 (3)	152

Symmetry code: (i) 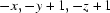. *Cg*1 and *Cg*2 are the centroids of the C2–C7 and C10–C15 rings, respectively.

**Fig. 1.**
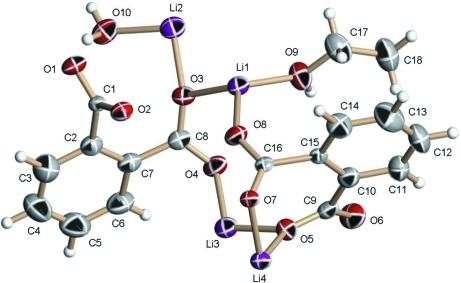
The asymmetric unit of the title compound, showing the atom-labelling scheme. Displacement ellipsoids are drawn at the 50% probability level and H atoms are shown as small spheres of arbitrary radii.

**Fig. 2.**
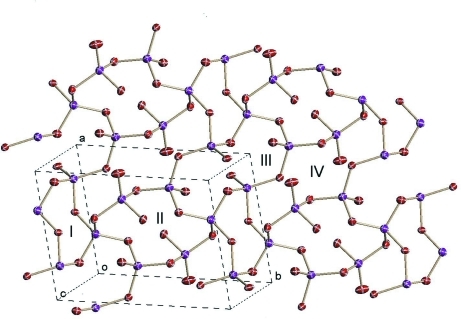
Crystal packing of the title compound, showing the two-dimensional network parallel to the ab plane. For clarity, hydrogen and carbon atoms are omitted.

**Fig. 3.**
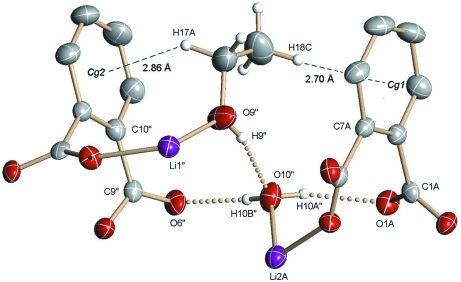
View of O—H···O and the facial C—H···π hydrogen bonds. Dashed lines indicate the vectors from the methylene (C17) and methyl (C18) groups to centroids Cg2 (C10—C15) and Cg1 (C2—C7), respectively.

## supplementary crystallographic information

### Comment

Metal-organic polymers are widely studied for their properties arising from the presence of a rigid framework with open channels and cavities. Frequently, such coordination polymers are constructed from metal ions and polycarboxylate linkers; however, systems containing main group elements are little explored so far (Łyszczek *et al.*, 2008; García-Zarracino *et al.*, 2008; Chae *et al.*, 2004; García-Zarracino *et al.*, 2003; García-Zarracino & Höpfl, 2004).

We describe herein the crystal structure of dilithium phthalate solvate [(C_8_H_4_O_4_)_2_Li_4_(H_2_O)(C_2_H_5_OH)]*_n_*. The asymmetric unit of the title compound contains two benzene-1,2-dicarboxylate ligands, four crystallographically independent lithium ions, an ethanol molecule and a water molecule. The four lithium ions have distorted tetrahedral coordination environments as shown in Fig. 1. The O—Li—O bond angles range from 96.90 (15) to 127.86 (19)°. The Li—O bond lengths vary from 1.901 (4) to 2.009 (4) Å, whereby it is interesting to note that the Li—O(H)Et bond is significantly shorter [1.910 (4) Å] than the remaining bonds, the only exception being Li—O1 [1.901 (4) Å]. Coordination of the crystallographically independent Li centers through bridging-bidentate benzene-1,2-dicarboxylate groups generates a two-dimensional network parallel to the *ab *plane (Fig. 2). In this two-dimensional coordination polymer four different Li_n_O_n _ring structures can be identified: I (n= 4), II, III (n =6) and IV (n=8). It is notheworthy to mention that the COO groups in the benzene-1,2-dicarboxylate ligands adopt different coordination modes and torsion angles with respect to the corresponding aromatic ring plane: µ_3_ and -72.2 (3)° for C(1)OO, µ_4_ and -24.9 (3)° for C(8)OO, µ_3_ and 21.0 (3)° for C(9)OO, µ_4_ and 88.8 (2)° for C(16)OO.

Packing is further stabilized by ethanol and water molecules which are coordinated to Li(1) and Li(2), respectively. Both solvent molecules display O—H···O and C—H···π hydrogen bonds (Table 1, Fig. 3). The distances from the methylene (C17) and methyl (C18) groups to centroids *Cg*2 (C10—C15) and *Cg*1 (C2—C7) are 3.584 (3) and 3.572 (3) Å, respectively (Hunter, 1994; Desiraju, 1991).

### Experimental

Single crystals  were obtained by slow evaporation of a solution (EtOH, 15 ml) containing benzene-1,2-dicarboxylic acid (0.50 g, 3.0 mmol) and lithium hydroxide dihydrate (0.36 g, 6.0 mmol).

### Refinement

Aromatic and aliphatic H atoms were positioned geometrically and constrained using the riding-model approximation [*C*-H_aryl_ = 0.93 Å, *U*_iso_(H_aryl_)= 1.2 *U*_eq_(C); *C*-H_methylene_ = 0.97 Å, *U*_iso_(H_methylene_) = 1.2 *U*_eq_(C); *C*-H_methyl_ = 0.96, *U*_iso_(H_methyl_) = 1.5 *U*_eq_(C)]. Atoms bonded to O (H9, H10A and H10B), were located by difference Fourier maps. Their coordinates were refined with a distance restraint O—H = 0.84 Å and [*U*_iso_(H) = 1.5 *U*_eq_(O)].

### Crystal data

**Table:**

[Li_4_(C_8_H_4_O_4_)_2_(C_2_H_6_O)(H_2_O)]	*Z* = 2
*M**_r_* = 420.07	*F*(000) = 432
Triclinic, *P*1	*D*_x_ = 1.467 Mg m^−^^3^
Hall symbol: -P 1	Mo *K*α radiation, λ = 0.71073 Å
*a* = 7.5254 (7) Å	Cell parameters from 5811 reflections
*b* = 10.0538 (10) Å	θ = 2.3–27.5°
*c* = 13.5073 (13) Å	µ = 0.12 mm^−^^1^
α = 106.460 (2)°	*T* = 293 K
β = 91.185 (2)°	Plate, colourless
γ = 103.046 (2)°	0.49 × 0.36 × 0.06 mm
*V* = 950.84 (16) Å^3^	

### Data collection

**Table:**

Bruker SMART APEX CCD area-detector diffractometer	3735 independent reflections
Radiation source: fine-focus sealed tube	3110 reflections with *I* > 2σ(*I*)
graphite	*R*_int_ = 0.039
Detector resolution: 8.3 pixels mm^-1^	θ_max_ = 26.0°, θ_min_ = 1.6°
φ and ω scans	*h* = −9→9
Absorption correction: multi-scan (*SADABS*; Sheldrick, 1996)	*k* = −12→12
*T*_min_ = 0.945, *T*_max_ = 1.0	*l* = −16→16
10069 measured reflections	

### Refinement

**Table:**

Refinement on *F*^2^	Primary atom site location: structure-invariant direct methods
Least-squares matrix: full	Secondary atom site location: difference Fourier map
*R*[*F*^2^ > 2σ(*F*^2^)] = 0.054	Hydrogen site location: inferred from neighbouring sites
*wR*(*F*^2^) = 0.129	H atoms treated by a mixture of independent and constrained refinement
*S* = 1.09	*w* = 1/[σ^2^(*F*_o_^2^) + (0.0657*P*)^2^ + 0.0955*P*] where *P* = (*F*_o_^2^ + 2*F*_c_^2^)/3
3735 reflections	(Δ/σ)_max_ < 0.001
299 parameters	Δρ_max_ = 0.29 e Å^−^^3^
3 restraints	Δρ_min_ = −0.20 e Å^−^^3^

### Special details

**Table:**

Geometry. All e.s.d.'s (except the e.s.d. in the dihedral angle between two l.s. planes) are estimated using the full covariance matrix. The cell e.s.d.'s are taken into account individually in the estimation of e.s.d.'s in distances, angles and torsion angles; correlations between e.s.d.'s in cell parameters are only used when they are defined by crystal symmetry. An approximate (isotropic) treatment of cell e.s.d.'s is used for estimating e.s.d.'s involving l.s. planes.
Refinement. Refinement of *F*^2^ against ALL reflections. The weighted *R*-factor *wR* and goodness of fit *S* are based on *F*^2^, conventional *R*-factors *R* are based on *F*, with *F* set to zero for negative *F*^2^. The threshold expression of *F*^2^ > σ(*F*^2^) is used only for calculating *R*-factors(gt) *etc*. and is not relevant to the choice of reflections for refinement. *R*-factors based on *F*^2^ are statistically about twice as large as those based on *F*, and *R*- factors based on ALL data will be even larger.

### Fractional atomic coordinates and isotropic or equivalent isotropic displacement parameters (Å^2^)

**Table:**

	*x*	*y*	*z*	*U*_iso_*/*U*_eq_	
Li1	0.3218 (5)	0.6867 (3)	0.6127 (3)	0.0274 (7)	
Li2	0.1872 (5)	0.3690 (4)	0.4990 (3)	0.0287 (8)	
Li3	0.1436 (5)	0.9010 (3)	0.4401 (3)	0.0271 (7)	
Li4	0.4303 (5)	1.1686 (4)	0.5415 (3)	0.0277 (7)	
O1	0.51646 (19)	0.40347 (14)	0.33549 (11)	0.0311 (4)	
H9	0.0361 (13)	0.716 (3)	0.6978 (19)	0.047*	
O2	0.60250 (18)	0.63805 (14)	0.41291 (11)	0.0277 (3)	
O3	0.21664 (18)	0.55545 (14)	0.47330 (10)	0.0263 (3)	
O4	0.05081 (18)	0.70170 (14)	0.44799 (11)	0.0277 (3)	
O5	0.20599 (18)	1.05156 (15)	0.57359 (10)	0.0272 (3)	
O6	−0.02639 (18)	1.04904 (15)	0.67106 (11)	0.0313 (4)	
O7	0.60652 (17)	1.08648 (14)	0.60341 (10)	0.0251 (3)	
O8	0.48228 (19)	0.86333 (14)	0.60088 (11)	0.0286 (3)	
O9	0.1425 (2)	0.7081 (2)	0.71036 (12)	0.0434 (4)	
O10	0.1786 (2)	0.24140 (16)	0.35725 (13)	0.0355 (4)	
H10A	0.2810 (17)	0.269 (3)	0.3365 (19)	0.053*	
H10B	0.155 (4)	0.1522 (4)	0.345 (2)	0.053*	
C1	0.5022 (3)	0.5289 (2)	0.34916 (15)	0.0224 (4)	
C2	0.3625 (3)	0.5497 (2)	0.27672 (15)	0.0236 (4)	
C3	0.3998 (3)	0.5279 (2)	0.17410 (17)	0.0351 (5)	
H3	0.5051	0.4983	0.1531	0.042*	
C4	0.2845 (4)	0.5489 (3)	0.10264 (17)	0.0408 (6)	
H4	0.3124	0.5345	0.0342	0.049*	
C5	0.1273 (4)	0.5915 (2)	0.13316 (18)	0.0414 (6)	
H5	0.0482	0.6052	0.0852	0.050*	
C6	0.0877 (3)	0.6136 (2)	0.23443 (16)	0.0314 (5)	
H6	−0.0179	0.6433	0.2545	0.038*	
C7	0.2028 (3)	0.59242 (19)	0.30770 (15)	0.0227 (4)	
C8	0.1526 (2)	0.6171 (2)	0.41697 (15)	0.0220 (4)	
C9	0.1401 (3)	1.05967 (19)	0.65956 (15)	0.0226 (4)	
C10	0.2681 (3)	1.0844 (2)	0.75287 (15)	0.0254 (4)	
C11	0.2166 (3)	1.1396 (2)	0.85110 (17)	0.0360 (5)	
H11	0.1030	1.1615	0.8579	0.043*	
C12	0.3293 (4)	1.1626 (3)	0.93858 (18)	0.0446 (6)	
H12	0.2924	1.1998	1.0038	0.054*	
C13	0.4977 (4)	1.1299 (3)	0.92891 (18)	0.0453 (6)	
H13	0.5747	1.1450	0.9878	0.054*	
C14	0.5521 (3)	1.0751 (2)	0.83213 (17)	0.0348 (5)	
H14	0.6654	1.0525	0.8262	0.042*	
C15	0.4401 (3)	1.0531 (2)	0.74352 (15)	0.0237 (4)	
C16	0.5114 (2)	0.9964 (2)	0.64110 (15)	0.0214 (4)	
C17	0.1858 (3)	0.7394 (3)	0.81807 (18)	0.0484 (7)	
H17A	0.2974	0.8148	0.8391	0.058*	
H17B	0.2091	0.6552	0.8322	0.058*	
C18	0.0380 (4)	0.7851 (3)	0.8815 (2)	0.0621 (8)	
H18A	0.0126	0.8675	0.8671	0.093*	
H18B	0.0772	0.8085	0.9538	0.093*	
H18C	−0.0708	0.7087	0.8644	0.093*	

### Atomic displacement parameters (Å^2^)

**Table:**

	*U*^11^	*U*^22^	*U*^33^	*U*^12^	*U*^13^	*U*^23^
Li1	0.0269 (18)	0.0260 (18)	0.0314 (19)	0.0084 (14)	0.0015 (14)	0.0105 (15)
Li2	0.0232 (18)	0.0280 (18)	0.036 (2)	0.0073 (14)	−0.0001 (14)	0.0106 (15)
Li3	0.0244 (18)	0.0259 (17)	0.0326 (19)	0.0064 (14)	0.0026 (14)	0.0107 (15)
Li4	0.0256 (18)	0.0272 (18)	0.0328 (19)	0.0062 (14)	0.0019 (15)	0.0129 (15)
O1	0.0299 (8)	0.0243 (8)	0.0430 (9)	0.0112 (6)	0.0011 (7)	0.0123 (6)
O2	0.0221 (7)	0.0252 (7)	0.0357 (8)	0.0050 (6)	−0.0027 (6)	0.0096 (6)
O3	0.0260 (8)	0.0268 (7)	0.0299 (8)	0.0089 (6)	0.0005 (6)	0.0126 (6)
O4	0.0228 (7)	0.0253 (7)	0.0397 (9)	0.0102 (6)	0.0086 (6)	0.0131 (6)
O5	0.0222 (7)	0.0308 (8)	0.0277 (8)	0.0048 (6)	0.0016 (6)	0.0088 (6)
O6	0.0184 (8)	0.0384 (9)	0.0428 (9)	0.0116 (6)	0.0064 (6)	0.0167 (7)
O7	0.0162 (7)	0.0272 (7)	0.0345 (8)	0.0047 (6)	0.0019 (6)	0.0137 (6)
O8	0.0335 (8)	0.0217 (7)	0.0341 (8)	0.0109 (6)	0.0055 (6)	0.0101 (6)
O9	0.0273 (9)	0.0688 (12)	0.0372 (9)	0.0183 (8)	0.0039 (7)	0.0150 (8)
O10	0.0246 (8)	0.0303 (8)	0.0503 (10)	0.0066 (7)	0.0062 (7)	0.0095 (8)
C1	0.0175 (10)	0.0254 (10)	0.0279 (11)	0.0076 (8)	0.0073 (8)	0.0111 (8)
C2	0.0238 (10)	0.0194 (10)	0.0271 (11)	0.0040 (8)	0.0005 (8)	0.0071 (8)
C3	0.0369 (13)	0.0377 (12)	0.0337 (12)	0.0132 (10)	0.0081 (10)	0.0117 (10)
C4	0.0558 (16)	0.0444 (14)	0.0243 (12)	0.0123 (12)	0.0024 (11)	0.0132 (10)
C5	0.0521 (15)	0.0398 (13)	0.0336 (13)	0.0143 (12)	−0.0121 (11)	0.0113 (10)
C6	0.0301 (12)	0.0278 (11)	0.0365 (13)	0.0114 (9)	−0.0063 (9)	0.0071 (9)
C7	0.0223 (10)	0.0177 (9)	0.0280 (11)	0.0045 (8)	−0.0026 (8)	0.0071 (8)
C8	0.0154 (9)	0.0192 (9)	0.0305 (11)	0.0019 (7)	−0.0009 (8)	0.0077 (8)
C9	0.0212 (10)	0.0172 (9)	0.0317 (11)	0.0069 (8)	0.0034 (8)	0.0093 (8)
C10	0.0259 (11)	0.0226 (10)	0.0294 (11)	0.0067 (8)	0.0040 (8)	0.0097 (8)
C11	0.0340 (13)	0.0394 (13)	0.0346 (13)	0.0120 (10)	0.0089 (10)	0.0083 (10)
C12	0.0504 (16)	0.0543 (15)	0.0258 (12)	0.0130 (12)	0.0097 (11)	0.0061 (11)
C13	0.0450 (15)	0.0578 (16)	0.0295 (13)	0.0053 (12)	−0.0079 (11)	0.0136 (11)
C14	0.0294 (12)	0.0409 (13)	0.0337 (12)	0.0083 (10)	−0.0037 (9)	0.0110 (10)
C15	0.0243 (10)	0.0202 (10)	0.0270 (10)	0.0035 (8)	0.0006 (8)	0.0094 (8)
C16	0.0148 (9)	0.0247 (10)	0.0281 (11)	0.0085 (8)	−0.0033 (8)	0.0105 (8)
C17	0.0380 (14)	0.0637 (17)	0.0381 (14)	0.0000 (12)	0.0020 (11)	0.0162 (12)
C18	0.067 (2)	0.0632 (19)	0.0450 (16)	0.0023 (15)	0.0195 (14)	0.0077 (14)

### Geometric parameters (Å, °)

**Table:**

Li1—O1^i^	1.901 (4)	O10—H10A	0.84 (2)
Li1—O9	1.910 (4)	O10—H10B	0.840 (10)
Li1—O8	1.959 (4)	C1—C2	1.510 (3)
Li1—O3	1.993 (4)	C2—C3	1.385 (3)
Li2—O3	1.967 (4)	C2—C7	1.398 (3)
Li2—O10	1.970 (4)	C3—C4	1.376 (3)
Li2—O2^i^	1.986 (4)	C3—H3	0.9300
Li2—O4^ii^	1.998 (4)	C4—C5	1.379 (3)
Li3—O6^iii^	1.964 (4)	C4—H4	0.9300
Li3—O5	1.965 (4)	C5—C6	1.373 (3)
Li3—O7^iv^	1.970 (4)	C5—H5	0.9300
Li3—O4	2.002 (3)	C6—C7	1.394 (3)
Li4—O2^iv^	1.940 (4)	C6—H6	0.9300
Li4—O5	1.961 (4)	C7—C8	1.497 (3)
Li4—O7	1.995 (4)	C9—C10	1.498 (3)
Li4—O8^iv^	2.009 (4)	C10—C11	1.386 (3)
O1—C1	1.251 (2)	C10—C15	1.400 (3)
O1—Li1^i^	1.901 (4)	C11—C12	1.373 (3)
O2—C1	1.257 (2)	C11—H11	0.9300
O2—Li4^iv^	1.940 (4)	C12—C13	1.379 (4)
O2—Li2^i^	1.986 (4)	C12—H12	0.9300
O3—C8	1.258 (2)	C13—C14	1.377 (3)
O4—C8	1.261 (2)	C13—H13	0.9300
O4—Li2^ii^	1.998 (4)	C14—C15	1.384 (3)
O5—C9	1.261 (2)	C14—H14	0.9300
O6—C9	1.250 (2)	C15—C16	1.499 (3)
O6—Li3^iii^	1.964 (4)	C17—C18	1.493 (4)
O7—C16	1.255 (2)	C17—H17A	0.9700
O7—Li3^iv^	1.970 (4)	C17—H17B	0.9700
O8—C16	1.258 (2)	C18—H18A	0.9600
O8—Li4^iv^	2.009 (4)	C18—H18B	0.9600
O9—C17	1.413 (3)	C18—H18C	0.9600
O9—H9	0.841 (13)		
			
O1^i^—Li1—O9	105.47 (17)	C4—C3—C2	121.5 (2)
O1^i^—Li1—O8	103.97 (17)	C4—C3—H3	119.3
O9—Li1—O8	116.44 (18)	C2—C3—H3	119.3
O1^i^—Li1—O3	106.04 (16)	C3—C4—C5	119.6 (2)
O9—Li1—O3	112.98 (18)	C3—C4—H4	120.2
O8—Li1—O3	110.81 (17)	C5—C4—H4	120.2
O3—Li2—O10	101.37 (17)	C6—C5—C4	119.9 (2)
O3—Li2—O2^i^	112.47 (17)	C6—C5—H5	120.0
O10—Li2—O2^i^	110.56 (17)	C4—C5—H5	120.0
O3—Li2—O4^ii^	114.83 (17)	C5—C6—C7	121.2 (2)
O10—Li2—O4^ii^	105.75 (17)	C5—C6—H6	119.4
O2^i^—Li2—O4^ii^	111.15 (18)	C7—C6—H6	119.4
O6^iii^—Li3—O5	115.79 (17)	C6—C7—C2	118.77 (19)
O6^iii^—Li3—O7^iv^	100.67 (16)	C6—C7—C8	119.09 (18)
O5—Li3—O7^iv^	97.71 (16)	C2—C7—C8	122.15 (17)
O6^iii^—Li3—O4	116.55 (17)	O3—C8—O4	123.65 (18)
O5—Li3—O4	115.86 (17)	O3—C8—C7	118.68 (17)
O7^iv^—Li3—O4	106.30 (16)	O4—C8—C7	117.65 (17)
O2^iv^—Li4—O5	104.30 (17)	O6—C9—O5	123.40 (18)
O2^iv^—Li4—O7	127.86 (19)	O6—C9—C10	118.31 (17)
O5—Li4—O7	96.90 (15)	O5—C9—C10	118.29 (17)
O2^iv^—Li4—O8^iv^	107.63 (16)	O6—C9—Li3^iii^	43.92 (12)
O5—Li4—O8^iv^	123.76 (18)	C11—C10—C15	118.73 (19)
O7—Li4—O8^iv^	98.37 (15)	C11—C10—C9	119.75 (19)
C1—O1—Li1^i^	136.25 (17)	C15—C10—C9	121.52 (17)
C1—O2—Li4^iv^	129.06 (16)	C12—C11—C10	121.5 (2)
C1—O2—Li2^i^	123.07 (16)	C12—C11—H11	119.2
Li4^iv^—O2—Li2^i^	107.38 (16)	C10—C11—H11	119.2
C8—O3—Li2	142.29 (16)	C11—C12—C13	119.5 (2)
C8—O3—Li1	112.98 (15)	C11—C12—H12	120.3
Li2—O3—Li1	100.59 (15)	C13—C12—H12	120.3
C8—O4—Li2^ii^	119.14 (16)	C14—C13—C12	120.1 (2)
C8—O4—Li3	116.27 (15)	C14—C13—H13	120.0
Li2^ii^—O4—Li3	124.59 (15)	C12—C13—H13	120.0
C9—O5—Li4	129.30 (16)	C13—C14—C15	120.8 (2)
C9—O5—Li3	130.62 (16)	C13—C14—H14	119.6
Li4—O5—Li3	99.23 (15)	C15—C14—H14	119.6
C9—O6—Li3^iii^	109.88 (16)	C14—C15—C10	119.38 (19)
C16—O7—Li3^iv^	128.87 (15)	C14—C15—C16	117.46 (18)
C16—O7—Li4	106.10 (15)	C10—C15—C16	123.16 (17)
Li3^iv^—O7—Li4	121.08 (15)	O7—C16—O8	123.81 (18)
C16—O8—Li1	139.52 (17)	O7—C16—C15	116.96 (17)
C16—O8—Li4^iv^	106.87 (16)	O8—C16—C15	119.11 (17)
Li1—O8—Li4^iv^	110.21 (15)	O9—C17—C18	113.4 (2)
C17—O9—Li1	122.55 (17)	O9—C17—H17A	108.9
C17—O9—H9	110.9 (17)	C18—C17—H17A	108.9
Li1—O9—H9	125.6 (17)	O9—C17—H17B	108.9
Li2—O10—H10A	105.3 (19)	C18—C17—H17B	108.9
Li2—O10—H10B	121.2 (18)	H17A—C17—H17B	107.7
H10A—O10—H10B	109 (3)	C17—C18—H18A	109.5
O1—C1—O2	125.25 (18)	C17—C18—H18B	109.5
O1—C1—C2	116.45 (17)	H18A—C18—H18B	109.5
O2—C1—C2	118.11 (16)	C17—C18—H18C	109.5
C3—C2—C7	119.07 (19)	H18A—C18—H18C	109.5
C3—C2—C1	116.90 (18)	H18B—C18—H18C	109.5
C7—C2—C1	124.02 (17)		
			
O10—Li2—O3—C8	48.3 (3)	C7—C2—C3—C4	0.8 (3)
O2^i^—Li2—O3—C8	166.4 (2)	C1—C2—C3—C4	−177.7 (2)
O4^ii^—Li2—O3—C8	−65.1 (3)	C2—C3—C4—C5	−0.6 (4)
O10—Li2—O3—Li1	−158.66 (16)	C3—C4—C5—C6	0.5 (4)
O2^i^—Li2—O3—Li1	−40.6 (2)	C4—C5—C6—C7	−0.7 (3)
O4^ii^—Li2—O3—Li1	87.9 (2)	C5—C6—C7—C2	0.9 (3)
O1^i^—Li1—O3—C8	−159.63 (16)	C5—C6—C7—C8	−179.59 (19)
O9—Li1—O3—C8	85.3 (2)	C3—C2—C7—C6	−1.0 (3)
O8—Li1—O3—C8	−47.4 (2)	C1—C2—C7—C6	177.47 (18)
O1^i^—Li1—O3—Li2	37.91 (19)	C3—C2—C7—C8	179.55 (18)
O9—Li1—O3—Li2	−77.1 (2)	C1—C2—C7—C8	−2.0 (3)
O8—Li1—O3—Li2	150.12 (17)	Li2—O3—C8—O4	105.0 (3)
O6^iii^—Li3—O4—C8	105.3 (2)	Li1—O3—C8—O4	−46.0 (2)
O5—Li3—O4—C8	−113.2 (2)	Li2—O3—C8—C7	−76.6 (3)
O7^iv^—Li3—O4—C8	−5.9 (2)	Li1—O3—C8—C7	132.36 (18)
C9^iii^—Li3—O4—C8	128.77 (16)	Li2^ii^—O4—C8—O3	−59.5 (3)
O6^iii^—Li3—O4—Li2^ii^	−74.8 (2)	Li3—O4—C8—O3	120.4 (2)
O5—Li3—O4—Li2^ii^	66.7 (2)	Li2^ii^—O4—C8—C7	122.07 (19)
O7^iv^—Li3—O4—Li2^ii^	173.99 (16)	Li3—O4—C8—C7	−58.0 (2)
C9^iii^—Li3—O4—Li2^ii^	−51.4 (2)	C6—C7—C8—O3	156.66 (18)
Li4^iv^—Li3—O4—Li2^ii^	153.92 (16)	C2—C7—C8—O3	−23.8 (3)
O2^iv^—Li4—O5—C9	−58.5 (2)	C6—C7—C8—O4	−24.8 (3)
O7—Li4—O5—C9	73.5 (2)	C2—C7—C8—O4	154.65 (18)
O8^iv^—Li4—O5—C9	178.40 (18)	Li3^iii^—O6—C9—O5	−23.6 (3)
C16^iv^—Li4—O5—C9	161.14 (16)	Li3^iii^—O6—C9—C10	155.94 (17)
O2^iv^—Li4—O5—Li3	131.37 (17)	Li4—O5—C9—O6	137.2 (2)
O7—Li4—O5—Li3	−96.65 (16)	Li3—O5—C9—O6	−55.7 (3)
O8^iv^—Li4—O5—Li3	8.3 (2)	Li4—O5—C9—C10	−42.3 (3)
C16^iv^—Li4—O5—Li3	−9.01 (16)	Li3—O5—C9—C10	124.8 (2)
O6^iii^—Li3—O5—C9	111.4 (2)	Li4—O5—C9—Li3^iii^	120.91 (19)
O7^iv^—Li3—O5—C9	−142.81 (18)	Li3—O5—C9—Li3^iii^	−71.9 (2)
O4—Li3—O5—C9	−30.4 (3)	O6—C9—C10—C11	−20.6 (3)
C9^iii^—Li3—O5—C9	84.1 (2)	O5—C9—C10—C11	158.97 (19)
O6^iii^—Li3—O5—Li4	−78.7 (2)	Li3^iii^—C9—C10—C11	19.7 (4)
O7^iv^—Li3—O5—Li4	27.15 (17)	O6—C9—C10—C15	159.43 (18)
O4—Li3—O5—Li4	139.50 (18)	O5—C9—C10—C15	−21.0 (3)
C9^iii^—Li3—O5—Li4	−105.99 (16)	Li3^iii^—C9—C10—C15	−160.3 (3)
Li3^iii^—Li3—O5—Li4	−125.95 (13)	C15—C10—C11—C12	−0.6 (3)
Li4^iv^—Li3—O5—Li4	55.88 (16)	C9—C10—C11—C12	179.4 (2)
O2^iv^—Li4—O7—C16	112.4 (2)	C10—C11—C12—C13	−0.1 (4)
O5—Li4—O7—C16	−1.76 (19)	C11—C12—C13—C14	0.2 (4)
O8^iv^—Li4—O7—C16	−127.46 (16)	C12—C13—C14—C15	0.6 (4)
C16^iv^—Li4—O7—C16	−103.37 (14)	C13—C14—C15—C10	−1.3 (3)
O2^iv^—Li4—O7—Li3^iv^	−88.1 (3)	C13—C14—C15—C16	178.5 (2)
O5—Li4—O7—Li3^iv^	157.75 (15)	C11—C10—C15—C14	1.3 (3)
O8^iv^—Li4—O7—Li3^iv^	32.0 (2)	C9—C10—C15—C14	−178.69 (18)
C16^iv^—Li4—O7—Li3^iv^	56.14 (18)	C11—C10—C15—C16	−178.52 (18)
O1^i^—Li1—O8—C16	−113.1 (2)	C9—C10—C15—C16	1.5 (3)
O9—Li1—O8—C16	2.4 (3)	Li3^iv^—O7—C16—O8	−51.7 (3)
O3—Li1—O8—C16	133.4 (2)	Li4—O7—C16—O8	105.6 (2)
O1^i^—Li1—O8—Li4^iv^	91.69 (18)	Li3^iv^—O7—C16—C15	124.2 (2)
O9—Li1—O8—Li4^iv^	−152.80 (18)	Li4—O7—C16—C15	−78.46 (19)
O3—Li1—O8—Li4^iv^	−21.8 (2)	Li3^iv^—O7—C16—Li4^iv^	−59.9 (2)
O1^i^—Li1—O9—C17	36.1 (3)	Li4—O7—C16—Li4^iv^	97.41 (14)
O8—Li1—O9—C17	−78.6 (3)	Li1—O8—C16—O7	−166.9 (2)
O3—Li1—O9—C17	151.5 (2)	Li4^iv^—O8—C16—O7	−11.2 (2)
Li1^i^—O1—C1—O2	−3.7 (4)	Li1—O8—C16—C15	17.3 (3)
Li1^i^—O1—C1—C2	171.2 (2)	Li4^iv^—O8—C16—C15	172.99 (17)
Li4^iv^—O2—C1—O1	−168.20 (19)	Li1—O8—C16—Li4^iv^	−155.7 (3)
Li2^i^—O2—C1—O1	2.7 (3)	C14—C15—C16—O7	−87.3 (2)
Li4^iv^—O2—C1—C2	17.0 (3)	C10—C15—C16—O7	92.6 (2)
Li2^i^—O2—C1—C2	−172.08 (17)	C14—C15—C16—O8	88.8 (2)
O1—C1—C2—C3	−69.0 (2)	C10—C15—C16—O8	−91.3 (2)
O2—C1—C2—C3	106.2 (2)	C14—C15—C16—Li4^iv^	107.8 (5)
O1—C1—C2—C7	112.5 (2)	C10—C15—C16—Li4^iv^	−72.3 (6)
O2—C1—C2—C7	−72.2 (2)	Li1—O9—C17—C18	166.4 (2)

Symmetry codes: (i) −*x*+1, −*y*+1, −*z*+1; (ii) −*x*, −*y*+1, −*z*+1; (iii) −*x*, −*y*+2, −*z*+1; (iv) −*x*+1, −*y*+2, −*z*+1.

### Hydrogen-bond geometry (Å, °)

**Table:**

*D*—H···*A*	*D*—H	H···*A*	*D*···*A*	*D*—H···*A*
O9—H9···O10^ii^	0.84 (1)	1.95 (2)	2.767 (2)	165 (2)
O10—H10A···O1	0.84 (2)	1.97 (2)	2.764 (2)	156 (2)
O10—H10B···O6^ii^	0.84 (1)	1.98 (1)	2.797 (2)	163 (3)
C17—H17A···Cg2	0.97	2.86	3.584 (3)	132
C18—H18C···Cg1^ii^	0.96	2.70	3.572 (3)	152

Symmetry codes: (ii) −*x*, −*y*+1, −*z*+1.
